# The Role of Transcription Factors in Gonad Development and Sex Differentiation of a Teleost Model Fish—Guppy (*Poecilia reticulata*)

**DOI:** 10.3390/ani10122401

**Published:** 2020-12-15

**Authors:** Maciej Kamaszewski, Marek Skrobisz, Maciej Wójcik, Kacper Kawalski, Adrian Szczepański, Patryk Bujarski, Hubert Szudrowicz, Andrzej Przemysław Herman, Jakub Martynow

**Affiliations:** 1 Department of Ichthyology and Biotechnology in Aquaculture, Faculty of Animal Science, Warsaw University of Life Sciences-SGGW (WULS-SGGW), Ciszewskiego 8, 02-786 Warsaw, Poland; maro.skrobisz@gmail.com (M.S.); s203060@sggw.edu.pl (K.K.); adrian_szczepanski@sggw.edu.pl (A.S.); s203046@sggw.edu.pl (P.B.); hubert_szudrowicz@sggw.edu.pl (H.S.); s203069@sggw.edu.pl (J.M.); 2 The Kielanowski Institute of Animal Physiology and Nutrition, Polish Academy of Sciences, Instytucka 3, 05-110 Jabłonna, Poland; a.herman@ifzz.pl

**Keywords:** Dmrt1, estrogen receptor β, gonad development, guppy, histology of ovaries and testes, Sox9

## Abstract

**Simple Summary:**

The research was carried out to determine differences in gonadal development in female and male guppies (*Poecilia reticulata*) at the molecular level. Fish from the *Poeciliidae* family is an excellent model for gonadal differentiation studies due to their fertility and the occurrence of sexual dimorphism. Due to exceptional exposure of the aquatic environment to xenobiotics and other pollutants released into water, it becomes necessary to consider their influence on fish and other aquatic organisms’ reproductive capacity. To better understand the processes occurring during gonadal development in guppies, we conducted our research on animals of different ages: 1, 60, and 360 dph (day post-hatching). By examining the basic mechanisms of sex determination in guppies, it will be possible to identify disorders in gonadal development caused by exposure to pollutants in the water.

**Abstract:**

The guppy (*Poecilia reticulata*) is one of the world’s most popular ornamental fish. Due to lecithotrophic viviparous, it is commonly used in toxicological studies and environmental monitoring. This study aimed to investigate the molecular mechanisms of gonad development and differentiation during guppy ontogenesis. The study mainly focused on the role and localization of potential specific sex markers and transcription factors: Sox9, Dmrt1, Erβ. For histological analysis, guppies of both sexes were collected at 1, 60, and 360 dph (day post-hatching). The gonads morphology and immunohistochemistry detection of mentioned markers localization were performed. The expression of Sox9 protein was compared between sexes. Histological analysis revelated all types of male germinal cells in 60 dph guppy’s testes. Maturated oocytes were visible in the ovaries of 360 dph fish. The Sox9 expression varied in spermatocytes and spermatids, from cellular to nuclear localization, and was higher in ovaries. Dmrt1 was detected in all testes groups and 360 dph ovaries. The Erβ was observed in both sexes at 60 and 360 dph. For the first time, the localization of transcription factors in guppy during ontogenesis was traced. The Sox9 designation as a factor regulating the development of germinal cells in adult guppies may facilitate the analysis of xenobiotics’ influence on fish’s reproductive system.

## 1. Introduction

The guppy (*Poecilia reticulata*) is one of the world’s most widespread *Poeciliidae* fish and one of the most popular freshwater species of aquarium fish. It is easy to maintain and breed and is very resistant to suboptimal water conditions. Due to its plasticity, it has acclimatized in many habitats worldwide within tropical and subtropical climates, becoming an important invasive species in some regions. This species occurred natively in South America and was introduced to all continents except Antarctica. In some areas, such as the Deccan Peninsula [1] or Australia [2], it has become an invasive species that significantly affects the natural environment. Its prevalence in areas beyond its natural range is due, among others, to its documented use in the biocontrol of mosquito populations that transmit diseases such as zika, dengue, or malaria. The guppy, similarly to gambusia, which is also called mosquitofish, is a larvivorous fish and contributes to effective biocontrol methods of mosquito populations [3]. However, this species’ high fertility and ease of acclimatization make this fish often perceived as an invasive species and a threat to biodiversity [4].

Due to all these features, this species has been of interest to scientists for many years. Guppies have been recognized as model fish, especially in research related to genetics, ecology, behavior, and toxicology [5,6,7].

Moreover, the guppy belongs to the *Poeciliidae* family, and, like other fish in this family, it is a species characterized by lecithotrophic viviparousness, which makes it an interesting model for the study of oviprotection. An additional advantage of this species as a model fish is an apparent sexual dimorphism, visible in body size (the male is smaller than the female), intense pigmentation, and the presence of the gonopodium, i.e., the copulatory organ [8,9].

In the context of the growing ecotoxicological threat, related on the one hand to the increasing emission of pollutants to the environment, especially aquatic ecosystems, and on the other hand to the detection of new groups of xenobiotics in the environment, it becomes necessary to conduct research taking into account chronic exposure and assessing the impact on reproductive potential. The guppy has been used in xenobiotic toxicity model studies and environmental monitoring. Scientists point out that some groups of xenobiotics may disturb sex formation in the population and may negatively affect these fish’ fertility [9,10,11]. In mammals, sex is determined during fertilization by combining genetic factors of paternal and maternal origin. However, teleost’s gender differentiation process may be more complex. Gender determination in fish may be due to monogenic or polygenic systems, with the genetic factor being localized on the autosomes or sex chromosomes. The process of gender formation in teleost can be modified by exposure to several environmental factors, such as temperature changes, embryo exposure to sex hormones or pollutions [12,13]. Despite the generally known development of gonads in *Poeciliidae* fish during ontogenesis [14], it is necessary to thoroughly understand this species’ physiology, emphasizing the molecular mechanisms of gonad development and differentiation during ontogenesis. So far, the mechanisms of sex determination in fish are poorly understood, primarily that no obvious sex markers have been found [15]. In guppy, the occurrence of sex chromosomes was stated [16]. However, it turns out that gonad development usually results from the interaction and cooperation of many molecular mechanisms. In many vertebrates, including fish, *sex-determining region Y* (*sry*) can play a crucial role during testis development. Expression of the transcription factor *sox9*, followed by genes such as *amh* or *fgf9*, directs gonadal development to the path of testicle development [17].

Similarly, Webster et al. [18] stated that the increased expression of the transcription factor Dmrt1 indicates the male gonadal pathway development. In some fish species, *dmrt1* expression is specific only to male gonads, but the others express this gene in both testes and ovaries [18]. Studies on gender differentiation in medaka (*Oryzias latipes*) have shown that paralogies *dmrt1*: *dmrt1b (y)* [19] and *dmy* are located in the chromosome Y, and their function is equivalent to the gene *sry* in mammals [20]. Another factor that modulates the development of gonads in fish may be the estrogen receptor β, which belongs to the ligand-dependent group of receptors. Estrogen’s complex with a ligand-binding domain is involved in the estrogenic response, modulating the developmental gonadal pathways [9,21]. Mentioned transcription factors, Sox9, Dmrt1, and estrogen receptor β are essential proteins affecting the formation of both sexes in fish through various mechanisms and interaction pathways.

Therefore, the study aimed to describe the formation of sexual dimorphism, and the development of guppy gonads, emphasizing changes in the localization of selected transcription factors influencing the sex differentiation of fish. Due to the still unclear role of various transcription factors and other proteins in sex formation in this species, the aim was also to determine the changes in the Dmrt1, Sox9, and the estrogen receptor β (Erβ) expression during guppy ontogenesis, from hatching to 360 days after hatching.

## 2. Materials and Methods

This experiment was conducted in accordance with ethical principles and was approved by the 3rd Local Ethics Commission regarding Experiments in Animals at the Warsaw University of Life Sciences (WULS-SGGW) in Warsaw, Poland, no 70/2015.

### 2.1. Animals

The experiment was conducted at the Department of Ichthyology and Biotechnology in Aquaculture, Warsaw University of Life Sciences. Guppies (*Poecilia reticulata*) of both sexes in wild type, kept in our aquaria for three generations, were breed in five 50 L aquariums in a density of 0.5 fish per liter and distributed randomly. Fish were kept at water temperature 25 ± 1 °C, pH 7.1–7.4, in hard water for one year. During the experiment, the photoperiod was carried out in a ratio of 14 to 10 h day and night, respectively. Each aquarium was equipped with an internal sponge filter. Guppies were fed twice daily *ad libitum* with commercial Tetra Min food (Tetra Company, Melle, Germany). The pregnant females were transferred to 20 L spawning tanks with water parameters such as in main tanks.

### 2.2. Histological Samples Collection

All fish collected for analysis were selected randomly. For histological analysis, ten fish were collected on the 1 day after hatching (dph) and ten male and female guppies at the 60 day (the moment of observation of the first external sexual dimorphism features), and 360 days after hatching (adult fish). The fish were anesthetized with a solution of MS-222 (tricaine methanesulfonate, 3-amino benzoic acid ethyl ester, Sigma-Aldrich, St. Louis, MO, USA) and then measured with an accuracy of 0.01 mm using an electronic caliper (Topex, Warsaw, Poland) and weighed with an accuracy of 0.01 g using an electronic scale (Radwag, Radom, Poland). The average weight and body length of fish subjected to histological analysis are shown in Table 1. The fish was fixed in Bouin’s histological and immunohistochemical analysis solution and after dehydration in a gradient of ethanol (50%, 70%, 80%, 90%, 96%, 99.8%), cleared in xylene, and embedded in paraffin.

### 2.3. Histological Analysis

Histological slides (5 µm thick) were made with a Leica RM2025 microtome (Leica Microsystems, Nussloch, Germany). The obtained cross-sections were stained with the hematoxylin-eosin (H/E) (Avantor Performance Materials Poland S.A., Gliwice, Poland) method according to the standard staining procedure to assess the stage of development of gonads and for the sex determination of examined fish. The developmental stages of oocytes have been evaluated using the method described by Lambert [22]. The oocyte diameter is presented as a size distribution by class according to the methodology described by Koya et al. [23].

### 2.4. Immunohistochemical Analysis

The Dmrt1, estrogen receptor β (Erβ), and Sox9 proteins, immunohistochemical staining was performed following the methodology described by Kamaszewski et al. [17,24]. The histological preparations obtained were deparaffinized in xylene and then rehydrated in a gradient of ethanol. The activity of endogenous peroxidase was blocked by 10 min incubation of the specimens with a 3% solution of hydrogen peroxide. The histological slides were then rinsed with Tris buffer (pH 8.0) (T-6664, Sigma). Incubation with primary antibodies was carried out in a humid chamber according to the parameters shown in Table 2.

The visualization was performed using the EnVision + System–HRP (DAKO, Glostrup, Denmark) according to the manufacturer’s instruction. Cell nuclei were stained with the Harris hematoxylin. During the immunohistochemical reaction, the negative control, not incubated with the primary antibody, was used. Subsequently, the specimens were dehydrated in a gradient of ethanol, cleared with xylene, and mounted with DPX. (Sigma-Aldrich, St. Louis, MO, USA).

### 2.5. Microscopic Observations

Microscopic observations of histological and immunohistochemical slides were carried out using the Nikon Eclipse 90i microscope with Nikon Digital Sight DS-U1 camera (Nikon Corporation, Tokyo, Japan). Pictures were prepared using the NIS-Elements AR 2.10 software (Nikon Corporation, Tokyo, Japan).

### 2.6. Western Blot Analysis

The protein concentrations were isolated using the Mammalian Cell Lysis Kit (Sigma-Aldrich, USA). The value of Sox9 concentrations was normalized to total protein content in each sample assayed using the Bradford method (reagent solution cat. num. 110306, Merck, KGaA, Darmstadt, Germany). The appropriate volume of molecular grade water (Sigma-Aldrich, USA) was added to a sample containing 50 μg of total protein to bring the total sample volume to 20 μL. Next, 19 μL of Laemmli buffer (Sigma-Aldrich, USA) and 1 μL of β–mercaptoethanol (Sigma-Aldrich, USA) was added. Such mixtures were boiled for 3 min. Electrophoresis was then performed in the presence of a molecular weight marker (Spectra Multicolor Broad Range Protein Ladder, Thermo Fisher Scientific Inc., Waltham, MA, USA). Denatured samples and molecular weight standards were loaded onto 4% to 12% polyacrylamide gels and subjected to electrophoresis in a Tris-glycine running buffer using the Protean II xi Cell (Bio-Rad Laboratories, Inc., Hercules, CA, USA) according to the manufacturer’s instructions. After electrophoresis, proteins were transferred in Tris-glycine blotting buffer to polyvinylidene difluoride membranes (Immobilon-P [0.45 μm], Merck KGaA, Darmstadt, Germany) using the Trans-Blot SD Semi-Dry Transfer Cell (Bio-Rad Laboratories, Inc., Hercules, CA, USA) for 40 min at 20 V. The membranes were blocked for 1 h at room temperature in blocking buffer made up of bovine serum albumin fraction V (Sigma-Aldrich, USA) dissolved in Phosphate-buffered saline (pH 7.5) with 0.1% Tween-20 (PBST) (Sigma-Aldrich, USA). After blocking, membranes were incubated overnight at 4 °C with primary antibodies rabbit polyclonal Sox9 antibody (cat no. sc-20095, Santa Cruz Biotechnology Inc., Dallas, TX, USA) dissolved in blocking buffer at dilution 1:300. After incubation, membranes were washing three times and incubated with the following secondary horseradish peroxidase (HRP) conjugated antibodies: bovine anti-rabbit immunoglobulin G (IgG)-HRP (cat no. sc-2379, Santa Cruz Biotechnology Inc., Santa Cruz, CA, USA) dissolved in blocking buffer at a dilution of 1:5000. After washing (3 × 10 min.), the membranes were visualized using Clarity™ Western ECL Substrate (Bio-Rad) and imaged using the ChemiDoc MP Imaging system (Bio-Rad) and ImageLab software (version 5.1, Bio-Rad). Densitometric analysis of the Western Blot image was performed using ImageJ 1.50b software (Wayne Rasband, National Institute of Health, Bethesda, MD, USA).

## 3. Results

### 3.1. Gonadal Differentiation

**Figure 1:**
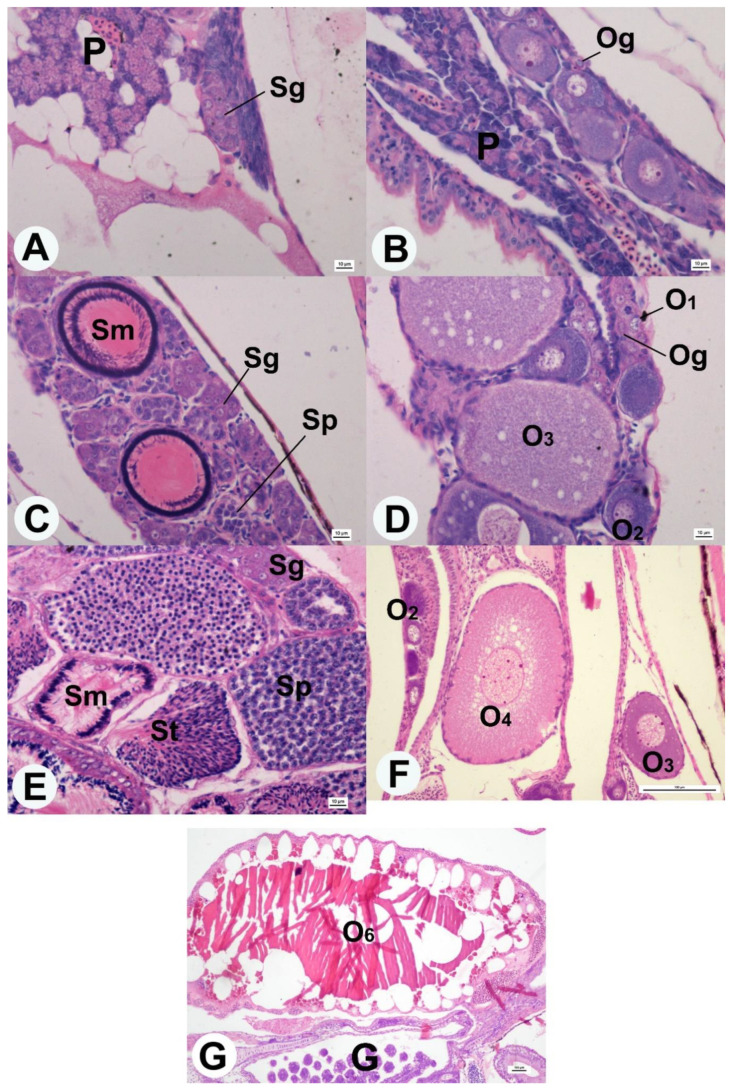
The cross-section of guppy gonad stained hematoxylin-eosin in age: 1 dph (**A**,**B**), 60 dph (**C**,**D**), and 360 dph (**E**–**G**). Localization of spermatogonia (Sg), spermatocytes (Sp), spermatids (St), and spermatozoa (Sm) in the cross-section of the testis (**A**,**C**,**E**). Localization of oogonia (Og) and oocytes stages: O_1_, O_2_, O_3_, O_4_, O_6_ in cross-section of ovaries (**B**,**D**,**F**,**G**). P—pancreas; G—embryo’s gills. Scale bars: (**A**–**E**)—10 μm; (**F**,**G**)—100 μm.

Guppies at 1-day post-hatching (dph) did not show characteristic external sexual dimorphisms such as male gonopodium or typical body pigmentation. Histological analysis of longitudinal sections by the fish revealed morphologically diversified gonads on the ovaries and testes. The testes showed growing clusters of germinal cells—spermatogonia (Figure 1A), surrounded by somatic cells. On the other hand, oogonia were observed in guppy ovaries among germinal cells, numerous oocytes in stages O_1_ (chromatin-nucleolus stage), O_2_ (peri-nucleolus stage I), and O_3_ (peri-nucleolus stage II) in early stages of development (Figure 1B).

Fish at 60 dph showed an advanced stage of gonad development. In males of this age, gonopodium development and a characteristic body coloration were observed, indicating that the fish have reached sexual maturity. Histological analysis of guppy testicles revealed in the parenchyma the presence of all types of germinal cells at various development stages: spermatogonia, spermatocytes, spermatids, and spermatozoa (Figure 1C). Moreover, seminal tubules were visible in the nucleus’s spatial structure, in which maturing spermatids and sperm cells have been observed (Figure 1C). In guppy ovaries, as in 1 dph, oogonia and oocytes were observed in the O_1_, O_2,_ and O_3_ stages. Due to the large number of lipid droplets distributed over the oocytes’ entire cross-section, some oocytes were classified into O_4_ (oil drop stage) (Figure 1D).

Guppies at 360 dph were sexually matured fish. Both the testes and the ovaries were significantly enlarged and occupied a significant part of the cross-section through the fish’s body cavity. In male gonadal sections, numerous seminal tubules filled with mature spermatozoids in the lumen and germinal cells at various development (spermatogonia, spermatocytes, and spermatids) were found in the tubules (Figure 1E). In the ovaries, in addition to oogonia and oocytes, at the stages found in the earlier stages of development (O_1_, O_2_, O_3_, O_4_), oocytes at other stages of development were also observed: O_5_ (primary yolk stage), O_6_ (secondary yolk stage), O_7_ (tertiary yolk stage) and O_8_ (maturation stage) (Figure 1F,G). In some of the analyzed females, the presence of embryos at various development stages was also found.

**Figure 2:**
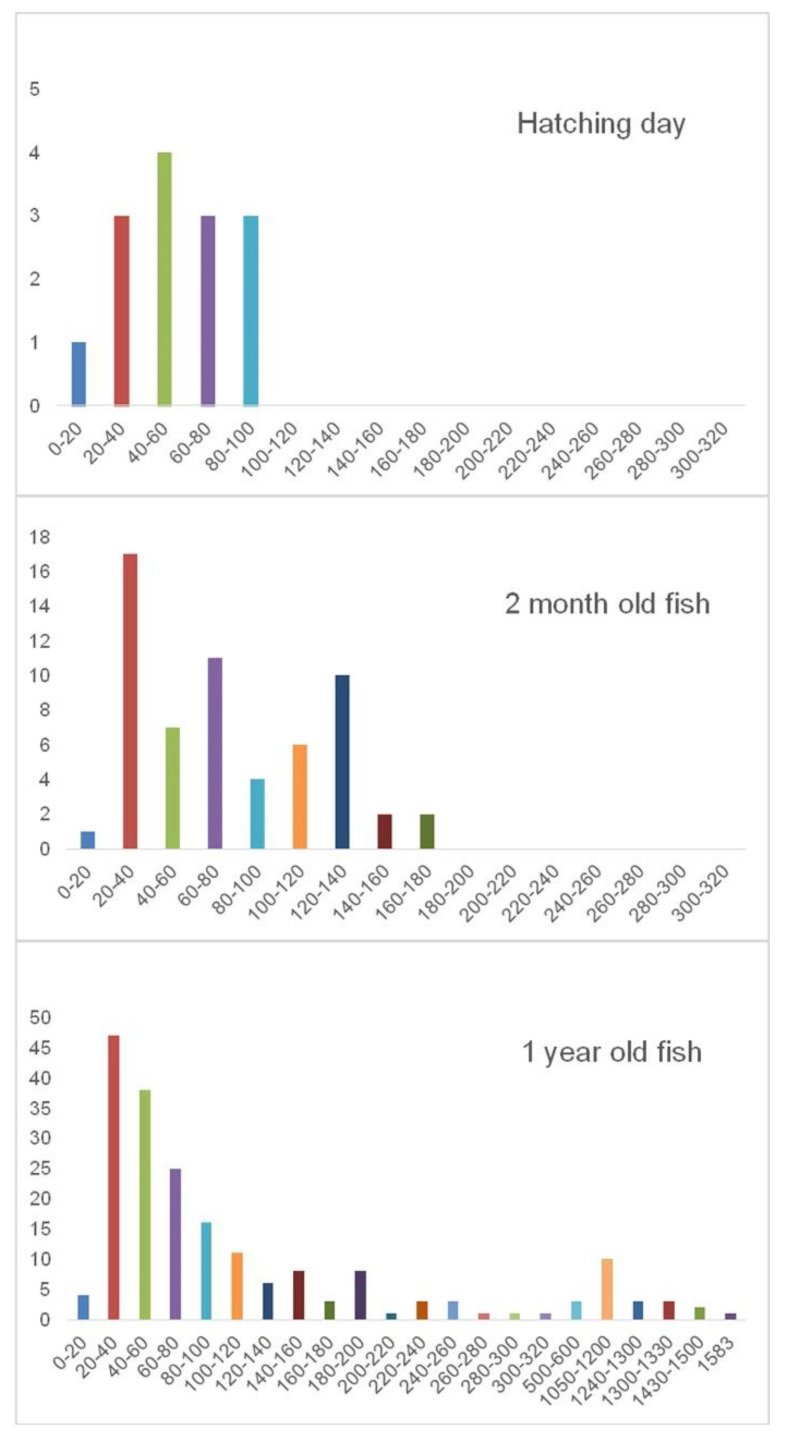
Distribution of guppy oocyte diameter on age 1, 60, and 360 dph. Vertical axis—number of oocytes, horizontal axis—oocyte diameter classes (µm).

Histomorphometric analysis of oocyte diameter distribution in the examined female guppies showed that in the ovary of 1 dph fish, oocytes with a diameter not exceeding 100 μm were observed, which indicates the presence of oocytes at the peri-nucleolus stage. In fish aged 60 dph, numerous oocytes 120–140 μm in diameter and larger were found in the ovary, indicating the presence of peri-nucleolus stage oocytes and an oil drop stage in the ovarian parenchyma. In 360 dph fish, the diameter distribution indicates oocytes’ presence at each stage of development (Figure 2).

### 3.2. Immunohistochemistry Detection of Transcription Factors Regulating Gonad Development

**Figure 3:**
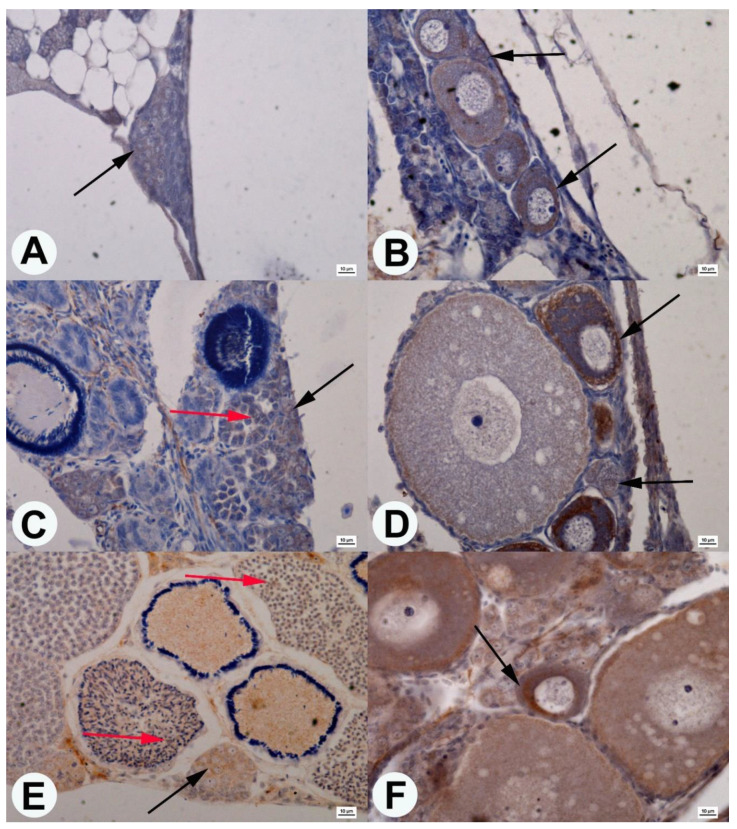
Cross-section of guppy testis (**A**,**C**,**E**) and ovaries (**B**,**D**,**F**) in age 1 dph (**A**,**B**), 60 dph (**C**,**D**), and 360 dph (**E**,**F**). Localization of the Sox9 protein (black arrow) in the cytoplasm of spermatogonia (**A**,**C**,**E**), oogonia (**B**,**D**), and oocyte in different stages (**B**,**D**,**F**). Localization of the Sox9 protein (red arrow) in the nucleus of spermatocytes and spermatids (**C**,**E**). Anti-Sox9 antibody staining. Scale bars: 10 μm.

Immunohistochemical analysis of fish gonads revealed Sox9-positive areas in both ovaries and testes from all studied age classes of guppies. A weak Sox9-positive reaction in the cytoplasm of spermatogonia was observed in the 1 dph aged fish testes (Figure 3A). Additionally, in the ovary, the Sox9 positive areas were observed in the cytoplasm of oogonia and oocytes (Figure 3B).

In males at 60 dph, Sox9 positive areas in the cytoplasm of spermatogonia were observed. On the other hand, in spermatocytes, a weak Sox9 reaction was observed in the nucleus, and a slightly colored cytoplasm around the nucleus (Figure 3C). In the ovary of the 60 dph guppies, the Sox9 protein was found in the cytoplasm of oogonia and oocytes (Figure 3D).

In guppy testes at the age of 360 dph, Sox9 positive areas were found in the cytoplasm of spermatogonia, while in the area of the cell nucleus—spermatocytes and spermatids (Figure 3E). In 360 dph females, as in the earlier development stage, Sox9 positive areas were observed in the cytoplasm of oogonia and oocytes (Figure 3F).

**Figure 4:**
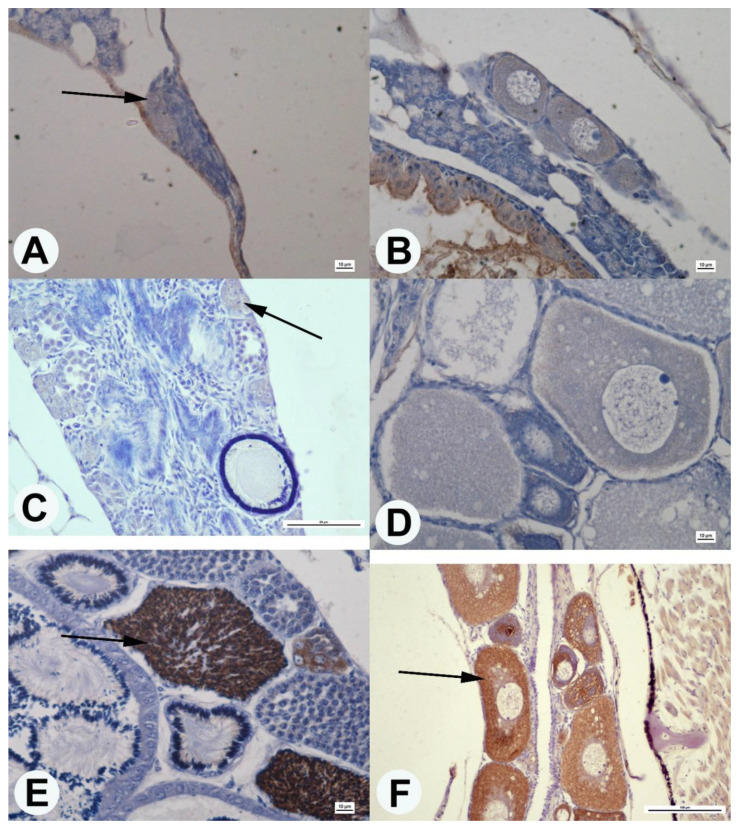
Cross-section of guppy testis (**A**,**C**,**E**) and ovaries (**B**,**D**,**F**) in age 1 dph (**A**,**B**), 60 dph (**C**,**D**), and 360 dph (**E**,**F**). Localization of the Dmrt1 protein (black arrow) in the cytoplasm of spermatogonia (**A**,**C**,**E**), spermatids (**E**), and oocyte in ovaries fish in age 360 dph. Anti-Dmrt1 antibody staining. Scale bars: (**A**,**B**,**D**,**E**)—10 μm; (**C**)—50 μm; (**F**)—100 μm.

The Dmrt1 protein was observed in testes of guppies ranging from 1 to 360 dph. Dmrt1 positive areas were located in spermatogonia’s cytoplasm and the nucleus of spermatids (Figure 4). No Dmrt1 positive areas were observed in the ovaries of guppies at 1 and 60 dph. Only in the ovaries cross-sections of fish at 360 dph, dark colors of the oocyte cytoplasm were observed that indicate the Dmrt1 protein (Figure 4).

**Figure 5:**
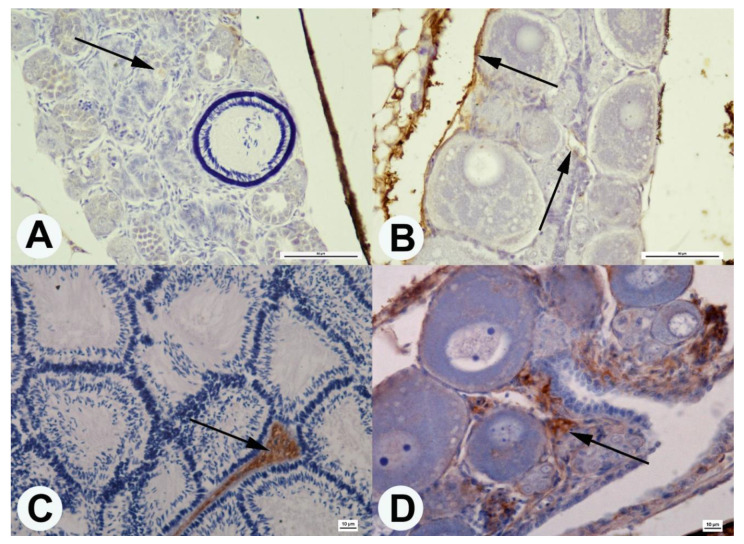
Cross-section of guppy testis (**A**,**C**) and ovaries (**B**,**D**) in age 60 dph (**A**,**B**) and 360 dph (**C**,**D**). Localization of the Erβ protein (black arrow) in the endothelium of blood vessels and interstitial tissue of gonads. Anti- Erβ antibody staining. Scale bars: (**A**,**B**)—50 μm; (**C**,**D**)—10 μm.

The estrogen receptor β protein was observed in both male and female gonads at 60 and 360 dph. In the testes, the estrogen receptor β was located in the endothelium of some blood vessels (Figure 5). On the other hand, in the ovaries, this protein’s presence was found in the endothelium of blood vessels and the gonadal interstitial tissue (Figure 5).

### 3.3. Western Blot Analysis

**Figure 6:**
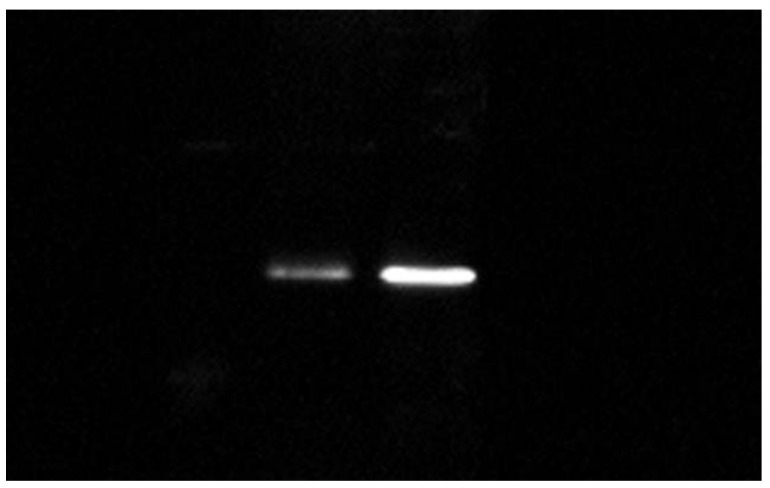
Western blot analysis of the Sox9 protein expression in testes (left) and ovaries (right).

The Sox9 protein expression was detected on the ovaries and testes. The densitometric analysis of obtained bands showed a significantly increased expression of Sox9 in ovaries compared to testes, and the expression was approximately three times higher (4741.598 to 1374.698, respectively) (Figure 6).

## 4. Discussion

In Gonochorist teleost, which includes guppy, many gonads development and sex differentiation mechanisms were found. As indicated by Devlin and Nagahama [12], environmental factors, such as temperature or xenobiotics acting directly in biochemical pathways that regulate the processes of differentiation and maturation of gonads, e.g., transcription factors, steroidogenic enzymes, receptors, may play a significant role in fish sex determination [25,26]. As Pettersson et al. [27] stated, the guppy is a gonochorist species for which natural is evenly sex distribution in the population. Moreover, it was found that in this species, sex is genetically determined. Differentiation of gonadal development into male or female begins during embryogenesis and ends with adolescence before the third month of life [22]. The guppy is the first organism to show that the inheritance of genes related to coloration is associated with the Y chromosome [7]. This is an essential feature of sexual dimorphism in this species. In the present research, it has been found that guppies’ sex can be distinguished using histological techniques already on the day of hatching. Koya et al. [14] showed that mosquitofish (*Gambusia affinis*), a species related to the guppy, starts meiotic divisions a few days before birth, which results in the formation of oocytes in the ovaries. Therefore, on the day of birth, the male and female gonads distinguish is possible.

Similarly, in the examined fish, with histological techniques, at the 1 dph, the gonads distinguished into the ovaries and testes were stated. Similarly, as in mosquitofish [14], at this stage of development, the female guppy gonad was characterized by a more advanced development stage than the male gonad, resulting from the presence of oogonia and oocytes at the stage O_1_, O_2_ and O_3_. Whereas only mitotically dividing spermatogonia was observed in the testicle.

In females at 60 dph, besides the stages O_1_, O_2_ and O_3_ observed in the 1 dph, the oil drop stage was spotted (O_4_). During this stage, an increased accumulation of oocytes in the cytoplasm lipids and more even distribution in the cytoplasm volume. Moreover, the oocyte diameter increases significantly at the oil drop stage, often above 0.3 mm [28]. As Koya et al. [23] showed, the constant presence of oocytes in the peri-nucleolus and oil-droplet stages is characteristic during gambling’s ovarian cycle. This may indicate that the researched female guppies have not yet reached full sexual maturity enabling the course of pregnancy, and oocyte maturation scarcely began in the gonad. The vitellogenesis process, during which the yolk is deposited in the oocytes’ cytoplasm, correlates with a significant diameter increase of the oocytes [23]. According to Lambert [22], during the oocyte’ development, from the oil drop stage (O_4_) until completion of the yolk accumulation in the tertiary yolk stage (O_7_), which increases the diameter of the oocytes even over five times. Observations of the histological structure of the studied fish ovaries confirm that in this species, the end of the juvenile phase of females is longer, and as indicated by Tian et al. [9], lasts up to three months of age. Unlike the female gonad, changes in guppies’ testes at the age of 60 dph indicate sexual maturity. Spermatogonia and spermatocytes at different stages of meiotic divisions were observed on the male gonad cross-sections.

Moreover, spermatids and spermatozoa in the forming seminal tubules were also observed in most researched fish. A similar testicular development stage was observed in another oviparous fish—the gambusia [14]. Moreover, in males at 60 dph, the external features of sexual dimorphism, i.e., characteristic body pigmentation and transformation of the anal fin into a copulatory organ—gonopodium were observed. These anatomical changes are described in guppies during the first three months of fish life [9] and can be a determinant of the achievement of functional gonadal maturity resulting from the appearance of sperm in the seminal tubules.

In mature fish (at the age of 360 dph), both in females and males, all types of germinal cells were observed, confirming the ongoing meiosis’s continuity. This is due to the short reproductive cycle and the aseasonality of guppy reproduction [28]. As Lambert [22] stated, all the oocyte development stages were observed in the female guppies’ ovaries. The distribution of oocyte diameters confirms that the tested species has a continuous reproduction strategy. Moreover, it suggests that some germinal cells are still developing and ready for fertilization after pregnancy, even in females during various pregnancy stages. On the other hand, in cross-sections of male guppies’ testes, an increase in the area occupied by spermatocytes’ cysts and seminal tubules’ number and surface was observed. Similarly, an increase in the proportion of spermatocytes and spermatids at the expense of spermatogonia was observed by Koya et al. [14] in cross-sections through the male gambusia gonad.

The development of gonads in vertebrates results from cell differentiation under the influence of transcription factors that take part and allow tissues to mature. As Kashimada and Koopman [29] stated, the *sex-determining region Y* (*sry*) is crucial in the process of male gonad formation in many mammal model species. The presence of the sry gene related to the *sox9* gene has not been found in many fish species. However, the *sox9* gene and protein are found in many Teleostei and non-Teleostei fish species, making it one of the most evolutionarily conserved factors determining the development of male gonads in vertebrates [26]. In the studied guppies, the Sox9 protein was found in both male and female gonads throughout all the experiment duration. However, from 60 dph in sexually mature males, during ontogenesis, a change in the localization of this protein in cells at the advanced stage of meiosis, i.e., spermatocytes and spermatids, from cytoplasmic to nuclear, was found. A similar change in the Sox9 protein location, which suggests the process of sexual maturation, was described by Kamaszewski et al. [17] in the gonads of Russian sturgeon (*Acipenser gueldenstaedtii*). These observations confirm the occurrence of the metabolic pathway also described in other vertebrates [30] and indicate that activation of this protein results in a change in the localization of this transcription factor from cytoplasmic to nuclear at the time of spermatogonia maturation. Therefore, this protein can be considered a potential sex marker and sexual maturity indicator.

Another transcription factor that plays a crucial role in male sex formation is the *dmrt1* gene [31]. As shown by Brunner et al. [32], its high expression is observed during the male gonad’s differentiation. The Dmrt1 protein is responsible for controlling male germinal cells’ proliferation and thus prevents the gonad’s feminization, preventing gene expression related to the ovarian formation [33,34]. The present study showed that the localization of the Dmrt1 protein in a guppy is observed in male germinal cells on the day of birth. On the other hand, the presence of Dmrt1 in the ovaries of female guppies at the age of 360 dph means that this protein cannot be considered a specific marker of the male sex. Expression of *dmrt1* in the female gonad has been reported in several species of fish, in presumptive oogonia at the immature stage of cod (*Gadus morhua*), maturing oocytes in older fish [35], and early differentiating oocytes of the medaka (*Oryzias latipes*) [31]. In the examined 1-year-old female guppies, Dmrt1 protein was found in the cytoplasm of oocytes in the peri-nucleolus, and oil-droplet stages may suggest an essential role of Dmrt1 in oogenesis [35].

Another factor that may influence teleosts’ sex may be the modulation of metabolic pathways regulated by various sex hormones, as in other vertebrates [36]. It has been found that estrogens in fish play a role in accumulating yolk in growing oocytes (vitellogenesis) and are involved in sex differentiation [37]. Nuclear receptors, including the estrogen receptor, are involved in estrogens’ response at the cellular level [38]. In guppies, the estrogen receptor β (Erβ) was observed in both the male and female gonads. In the examined fish of both sexes, Erβ showed a positive reaction within the endothelium of blood vessels, and only in the ovaries, the presence of Erβ positive areas in interstitial tissue was observed. This may suggest a more significant role of this receptor in determining the female sex by estrogens signal transduction, female sex hormones, and regulating the process of vitellogenesis. As indicated by [39], guppy’s estradiol content increases during vitellogenesis and drops abruptly after fertilization. The re-increase in this hormone’s content is found during advanced pregnancy when new oocytes are recruited. Examined females at 60 dph, only oocytes were observed in the ovary in which there is no yolk deposition in the cytoplasm (up to the stage O_4_). Therefore, a higher level of Erβ detection in the ovaries of the examined guppies was found on the last day of the experiment, when oocytes were also observed in the ovarian parenchyma at various deposition stages yolk plaques in the cytoplasm.

Based on the histological and immunohistochemical analyzes of the guppies, a potential sex marker used in toxicological studies and environmental monitoring was selected. As indicated by Tian et al. [9], guppies, despite the numerous advantages of this model species, should only be used in the adolescent period to study the effects of factors on the reproductive system. As males reach sexual maturity and development of gonopodium, they become insensitive to environmental chemicals. Therefore, it has been shown that only in the redistribution of Sox9 can find the type of expression dependent on sex, enabling the analysis of disorders of the reproductive system’s functioning due to xenobiotics’ influence. Erβ and Dmrt1 cannot be markers of sexual dysfunction in monitoring the impact of pollutants in a population because their expression may vary during maturation between sexes, and their expression may change at different stages of pregnancy. It has been shown in many fish species that xenoestrogens, including phytoestrogens, can influence sex determination [40,41]. Therefore, due to the susceptibility of Erβ to environmental factors, it is not recommended to use this protein as a marker of sex differentiation.

However, in the Sox9 protein case, despite the presence in fish both sexes, a change in localization from cytoplasmic to nuclear was observed in sexually mature males. As Berbejillo et al. [42] stated, Sox9 plays an essential role in the final stage of the nucleus’ differentiation and functioning; however, there were no stated differences in its expression between the nucleus ovary of Siberian sturgeon (*Acipenser baerii*). In the examined guppies, a threefold difference in Sox9 protein content was found between the male and female gonads. Therefore, this protein could be a potentially useful marker of gonadal dysfunction in sexually mature guppies.

## 5. Conclusions

The presented results are the first in which the transcription factor proteins’ localization during the model organism’s development—the guppy, was traced. Due to the differentiation of gonads into males and females on the day of delivery, this species can be used to analyze xenobiotics’ influence on the development of gonads and sex distribution in the population. However, gonadal development and sex differentiation in guppies are asynchronous. On the hatching day, female gonads development were more advanced than males. Conversely, at 60 dph, males are sexually matured, while females have no oocytes ready for fertilization. These changes are correlated with the appearance of external features of sexual dimorphism, which may facilitate the analysis of the effect of environmental pollution on the gender structure of the population. Additionally, the indication of Sox9 as a factor regulating the development of germinal cells in adult guppies may facilitate the analysis of disorders of the reproductive system of fish exposed to low environmental concentrations of various groups of xenobiotics in ecotoxicological studies.

## Figures and Tables

**Table 1:** Average body mass and length of collected fish collected for analysis.

Age and Sex	Body Mass (g)	Total Length (mm)
	Average	Average
1 dph, both sexes	0.007 ± 0.001	8.82 ± 0.69
60 dph, both sexes	0.043 ± 0.02	15.34 ± 2.32
360 dph, male	0.084 ± 0.039	21.96 ± 2.65
360 dph, female	0.15 ± 0.11	23.861 ± 5

**Table 2:** Primary antibodies and immunohistochemical staining conditions.

Primary Antibodies	Catalog Number, Manufacturer	Dilution	Incubation Conditions
Sox9 (H-90), rabbit, polyclonal	sc-20095, Santa Cruz Biotechnology, Dallas, TX, USA.	1:200	4 °C Overnight incubation
Estrogen receptor β (Er β), mouse, monoclonal	NCL-ER-beta, Leica Biosystems, Nussloch, Germany	1:200	4 °C Overnight incubation
Dmrt1 (A-9), mouse, monoclonal	sc-377167, Santa Cruz Biotechnology, Dallas, TX, USA.	1:200	4 °C Overnight incubation
